# Tribute to Dr. P. F. Eve

**Published:** 1878-02-20

**Authors:** W. F. Barr, E. M. Campbell

**Affiliations:** Committee; Abingdon, Va.; Committee; Abingdon, Va.


### TRIBUTE TO DR. P. F. EVE.

Abingdon, Va., Dec., 1878.

ABINGDON ACADEMY OF MEDICINE.

At a regular meeting of the Abingdon Academy of Medicine, the unexpected death of Paul F. Eve, M.D., professor of Operative and Clinical Surgery in the University of Nashville, and the Vanderbilt University, and an Honorary Fellow of the Abingdon Academy of Medicine, was announced. Drs. W. F. Barr and E. M. Campbell were appointed a Committee to report suitable preamble and resolutions on the death of Prof. Eve.

The Committee made the following report:

The Committee appointed to report a preamble and resolutions on the death of Paul F. Eve, M.D., of Nashville, Tenn., beg leave to offer the following report:

Of the distinguished men in medicine and surgery of the nineteenth century, few have occupied a more enviable position than Dr. Paul F. Eve, late professor in the Chair of Operative and Clinical Surgery, in the University of Nashville, and Vanderbilt University. Never obtrusive, his exalted merit attracted an attention almost universal; and judging from the many positions of trust he filled, and always to the fullest satisfaction, few men will be more missed than Dr. Eve. To know the man, was but to admire him, not only for his intrinsic merit as a man, but for his superior knowledge as a surgeon, and his remarkable success in his exalted position in his profession; therefore,

1. *Resolved, by the Abingdon Academy of Medicine*, that we deeply deplore the loss to his country, the profession and the world, of such a man as Prof. Paul F. Eve, M.D.

2. *Resolved*, That this Association will ever endeavor to emulate the exalted virtues, and the profound professional skill of one so eminent in all that constitutes the character of a good, noble, and generous man.

3. *Resolved*, That a copy of the above preamble and resolutions be furnished the family of the deceased; and also to the *Virginia Medical Monthly*, *Nashville Journal of Medicine and Surgery*, and the SOUTHERN MEDICAL RECORD, for publication

W. F. BARR, M.D.
E. M. CAMPBELL, M.D.
Committee.

ATLANTA MEDICO-CHIRURGICAL ASSOCIATION.—The officers elect for the above Society for the semi-annual term ending in June next, are as follows:

G. G. Crawford, M.D., President.
E. J. Roach, M.D., 1st Vice-President.
H. B. Lee, M. D., 2d Vice-President.
R. C. Word, M.D., Secretary.

The regular meetings are appointed on the first and third Friday evening of each month, at the office of Dr. J. J. Knott, until such time as a Hall be procured.

